# Organic-Solvent-Tolerant Carboxylic Ester Hydrolases for Organic Synthesis

**DOI:** 10.1128/AEM.00106-20

**Published:** 2020-04-17

**Authors:** Alexander Bollinger, Rebecka Molitor, Stephan Thies, Rainhard Koch, Cristina Coscolín, Manuel Ferrer, Karl-Erich Jaeger

**Affiliations:** a Institute of Molecular Enzyme Technology, Heinrich Heine University Düsseldorf, Jülich, Germany; b Bayer AG, Leverkusen, Germany; c Institute of Catalysis, Consejo Superior de Investigaciones Científicas, Madrid, Spain; d Institute for Bio- and Geosciences IBG-1, Biotechnology, Forschungszentrum Jülich GmbH, Jülich, Germany; University of Tartu

**Keywords:** *Alcanivorax borkumensis*, *Pseudomonas aestusnigri*, carboxylic ester hydrolases, high-throughput screening, polar organic solvent

## Abstract

Biocatalysis has emerged as an important tool in synthetic organic chemistry enabling the chemical industry to execute reactions with high regio- or enantioselectivity and under usually mild reaction conditions while avoiding toxic waste. Target substrates and products of reactions catalyzed by carboxylic ester hydrolases are often poorly water soluble and require organic solvents, whereas enzymes are evolved by nature to be active in cells, i.e., in aqueous rather than organic solvents. Therefore, biocatalysts that withstand organic solvents are urgently needed. Current strategies to identify such enzymes rely on laborious tests carried out by incubation in different organic solvents and determination of residual activity. Here, we describe a simple assay useful for screening large libraries of carboxylic ester hydrolases for resistance and activity in water-miscible organic solvents. We have screened a set of 26 enzymes, most of them identified in this study, with four different water-miscible organic solvents. The triglyceride tributyrin was used as a substrate, and fatty acids released by enzymatic hydrolysis were detected by a pH shift indicated by the indicator dye nitrazine yellow. With this strategy, we succeeded in identifying a novel highly organic-solvent-tolerant esterase from Pseudomonas aestusnigri. In addition, the newly identified enzymes were tested with sterically demanding substrates, which are common in pharmaceutical intermediates, and two enzymes from Alcanivorax borkumensis were identified which outcompeted the gold standard ester hydrolase CalB from Candida antarctica.

**IMPORTANCE** Major challenges hampering biotechnological applications of esterases include the requirement to accept nonnatural and chemically demanding substrates and the tolerance of the enzymes toward organic solvents which are often required to solubilize such substrates. We describe here a high-throughput screening strategy to identify novel organic-solvent-tolerant carboxylic ester hydrolases (CEs). Among these enzymes, CEs active against water-insoluble bulky substrates were identified. Our results thus contribute to fostering the identification and biotechnological application of CEs.

## Abstract (precis)

Major challenges hampering biotechnological applications of esterases include the requirement to accept nonnatural and chemically demanding substrates and the tolerance of the enzymes toward organic solvents which are often required to solubilize such substrates. We describe here a high-throughput screening strategy to identify novel organic-solvent-tolerant carboxylic ester hydrolases (CEs). Among these enzymes, CEs active against water-insoluble bulky substrates were identified. Our results thus contribute to fostering the identification and biotechnological application of CEs.

## INTRODUCTION

Enzymes are frequently used in biotechnology and are of high interest for many commercial applications (1–3). Besides the detergent, dairy, and baking industries, they are successfully applied in the fine-chemical and pharma sectors because of their superior stereo- and regioselectivity (1, 2, 4). This is reflected by a steadily growing market for enzymes and products thereof, as well as by industrial attempts to protect intellectual property in this field (5, 6). Indeed, the high demand has contributed to the fact that 2018 was named the Year of Biotechnology (7), due to the fact that private biotech companies raised more money in 2018 than in any previous year.

The combination of metagenomics and next-generation sequencing has resulted in the rapid accumulation of sequence data and, as a consequence, *in silico* predictions of numerous novel biocatalysts (8, 9). However, the vast majority of this sequence information is not validated experimentally in terms of confirmation of a proposed function and therefore is of limited use (10).

Hydrolases (EC 3) represent one of the most important class of enzymes for biocatalytic applications catalyzing a wealth of different hydrolysis reactions, amidations, kinetic resolutions, esterifications, polycondensations, and many other reactions (11). Among the hydrolases, carboxylic ester hydrolases (CEs) (EC 3.1.1), which catalyze the reversible hydrolysis of carboxylic ester bonds, have been found to have wide applications. This is why novel CEs are targets of screening programs, in which they are identified by different high-throughput screening systems, including halo formation on agar plates, chromogenic and fluorimetric methods, pH shift detection, fluorescence-activated cell sorting (FACS) techniques, microfluidic systems, mass spectroscopic analysis, and other systems (12, 13).

Enzymes of this class can be found in every living organism; however, marine hydrocarbonoclastic bacteria, also known as marine crude oil-degrading bacteria, have been shown to be a prolific source for biotechnologically relevant CEs (14). These bacteria live in close contact to alkanes (15), their preferred source of carbon and energy, some of which are organic solvents. Hence, it is reasonable to assume that crude oil-degrading bacteria may encode and produce organic-solvent-tolerant enzymes. The best studied example from this group of bacteria is Alcanivorax borkumensis SK2, with at least 12 different CEs with experimentally proven activity (16–19). In contrast, the crude oil-associated bacterium Pseudomonas aestusnigri VGXO14 (20) is almost unexplored with respect to CE activity, but its genome sequence hints at a number of CE-encoding genes (21).

Biotechnological applications of CEs and enzymes in general often require the biocatalyst to operate under nonnatural reaction conditions and accept artificial substrates rendering substrate promiscuity and enzyme tolerance for extreme pH, salt, and organic solvents a prerequisite for application. In organic synthesis in particular, substrates and/or products are usually not water soluble, thus requiring the presence of water-miscible organic solvents. Whereas a broad substrate specificity can (at least to a certain extent) be predicted from primary sequence information (17), it is still very difficult to predict solvent tolerance exclusively from primary sequence information. Furthermore, experimental data on solvent tolerance are usually obtained by measuring residual enzyme activities in buffer solutions after prior incubation in organic solvents. Preferably, both incubation and activity measurements should be performed in the presence of organic solvents.

In the present study, we describe a set of 25 CEs, 15 of which were newly identified in this study, from *A. borkumensis* and *P. aestusnigri*. Using a simple high-throughput assay, organic-solvent-tolerant CEs were found and tested for their ability to hydrolyze water-insoluble substrates. As a result, we report on novel CEs with broad substrate promiscuity and high organic solvent tolerance.

## RESULTS

### Cloning and expression of carboxylic ester hydrolases.

Mineral oil-degrading bacteria have been proven to be a prolific source of lipolytic enzymes (9, 22, 23). In this study, we focused on two marine hydrocarbonoclastic bacteria, namely, Alcanivorax borkumensis and Pseudomonas aestusnigri, and screened them for CEs. In total, we constructed a set of 26 different CEs (Table 1; see also Table S1 in the supplemental material) belonging to different families of bacterial lipolytic enzymes (24, 25) and showing an overall low sequence identity (Fig. S1). Eight of these CEs were first described by Martínez-Martínez et al. (17), one CE was identified by Hajighasemi et al. (18), one CE was identified recently by Bollinger et al. (26), and 15 CEs were newly identified in this study. Of the CEs used in this study, 16 CEs were recovered from genome libraries after naive screening; additionally, 9 CEs were identified through genome sequence searches. All CEs identified from genome sequences and 6 of the CEs recovered from genome libraries were cloned into pET-22b(+) high-level expression vectors (Table 1). The remaining 10 CEs obtained from library screens were cloned as genomic fragments into pCR-XL-TOPO vectors, resulting in mediocre expression levels (Tables 1 and S1). The set was completed by HZ lipase from Aneurinibacillus thermoaerophilus, which was previously described as organic solvent tolerant and thermostable (27, 28) and was thus used as a benchmark enzyme. In all cases, the presence of enzymatically active CEs was confirmed by hydrolysis of the substrate 4-nitrophenyl butyrate after heterologous expression in Escherichia coli BL21(DE3) (data not shown).

**TABLE 1:** Carboxylic ester hydrolases cloned and expressed in this study

Source organism	ID^*a*^	NCBI protein accession no.	Vector	Reference or source
Aneurinibacillus thermoaerophilus HZ	CE01	ADC84241.1	pET-22b(+)	27
Alcanivorax borkumensis SK2	CE02^*b*^	CAL15491.1	pET-22b(+)	17
	CE03^*b*^	CAL15643.1	pET-22b(+)	17
	CE04^*b*^	CAL16699.1	pCR-XL-TOPO	17
	CE05^*b*^	CAL17546.1	pCR-XL-TOPO	17
	CE06^*b*^	CAL17897.1	pCR-XL-TOPO	17
	CE07^*b*^	CAL17902.1	pET-22b(+)	This study
	CE08^*b*^	CAL18147.1	pCR-XL-TOPO	17
	CE09^*c*^	CAL17556.1	pET-22b(+)	This study
	CE10^*c*^	CAL18112.1	pET-22b(+)	17
	CE11^*c*^	CAL17943.1	pET-22b(+)	17
	CE12^*c*^	CAL16116.1	pET-22b(+)	19
Pseudomonas aestusnigri VGXO14	CE13^*b*^	WP_088275369.1	pET-22b(+)	This study
	CE14^*b*^	WP_088277870.1	pET-22b(+)	This study
	CE15^*b*^	WP_088277153.1	pET-22b(+)	This study
	CE16^*c*^	WP_088276085.1	pET-22b(+)	26
	CE17^*c*^	WP_088276582.1	pET-22b(+)	This study
	CE18^*c*^	WP_088273225.1	pET-22b(+)	This study
	CE19^*c*^	WP_088277509.1	pET-22b(+)	This study
	CE20^*c*^	WP_088273217.1	pET-22b(+)	This study
	CE21^*b*^	WP_088273788.1	pCR-XL-TOPO	This study
	CE22^*b*^	SEG59772.1	pCR-XL-TOPO	This study
	CE23^*b*^	WP_088274564.1	pCR-XL-TOPO	This study
	CE24^*b*^	WP_088275865.1	pCR-XL-TOPO	This study
	CE25^*b*^	WP_088273867.1	pCR-XL-TOPO	This study
	CE26^*b*^	ND^*d*^	pCR-XL-TOPO	This study

a Enzyme identifier (ID) used in this study.

b CEs identified by naive screening.

c CEs identified by genome mining.

d The coding sequence of this esterase was not determined (ND); the DNA fragment carried by the library clone was identical to Pseudomonas aestusnigri VGXO14 scaffold00001 NBYK01000001.1 positions 282473 to 286927 (see also Table S1).

### Screening of CEs for organic solvent tolerance.

**FIG 1:**
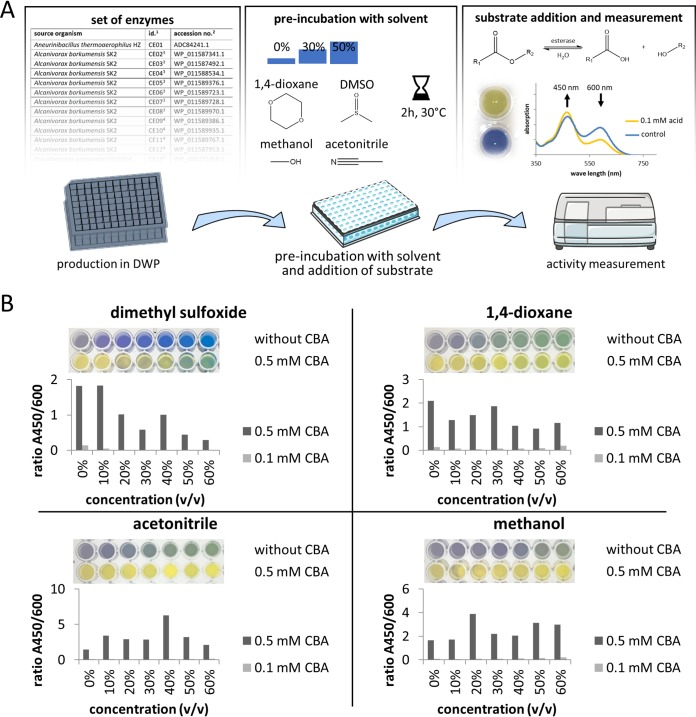
High-throughput identification of organic-solvent-tolerant CEs using the nitrazine yellow assay. (A) Workflow of the nitrazine yellow assay. Pictures of the 96-well plate and the plate reader were retrieved from Servier Medical Art, licensed under Creative Commons Attribution 3.0 (CC BY). (B) Nitrazine yellow (20 μg/ml) was mixed with different concentrations of organic solvent and potassium phosphate buffer (5 mM) titrated with potassium hydroxide solution (10 mM) until a neutral pH was reached (indicated by a light-green to blue color). After the addition of 2-chlorobenzoate (2-CBA), the pH shift was measured photometrically by determining the ratios of absorbance at 450 and 600 nm compared to a control without CBA.

Organic solvent tolerance of enzymes is usually determined by incubation at a defined solvent concentration for a limited time period (e.g., 30 min) and subsequent activity measurement in a buffer without solvent. Determination of enzymatic activity in the absence of organic solvent may give rise to false-positive results. We found that a pH indicator-based assay using nitrazine yellow dye (29) (Fig. 1A) yields reliable results in the presence of organic solvent concentrations up to 50% (vol/vol) (Fig. 1B). Four different water-miscible organic solvents were chosen based on their relevance for synthetic organic chemistry (30), namely, methanol, acetonitrile, dimethyl sulfoxide, and 1,4-dioxane, and were tested at two concentrations, 30% and 50% (vol/vol). To exclude enzymes, which are only active for a short time in the presence of organic solvents, a 2-h preincubation step was introduced before the substrate (tributyrin) was added to the reaction. After the addition of the substrate and incubation for 18 h, the ratio of the indicator absorptions at 450 and 600 nm, respectively, was determined. The absorption of a reaction mixture without substrate was subtracted. This is important when whole-cell extracts are used as in this study, which may contain intrinsic CEs active toward membrane lipids. Cloned CEs were expressed either at high levels from promoter P_T7_ in pET22b(+) or at a low level from their native promoters in a pCR-TOPO-XL-based genomic library. E. coli cells were perforated by treatment with polymyxin B, and cell lysates were transferred into assay plates semiautomatically using a 96-channel pipette (Platemaster; Gilson).

**FIG 2:**
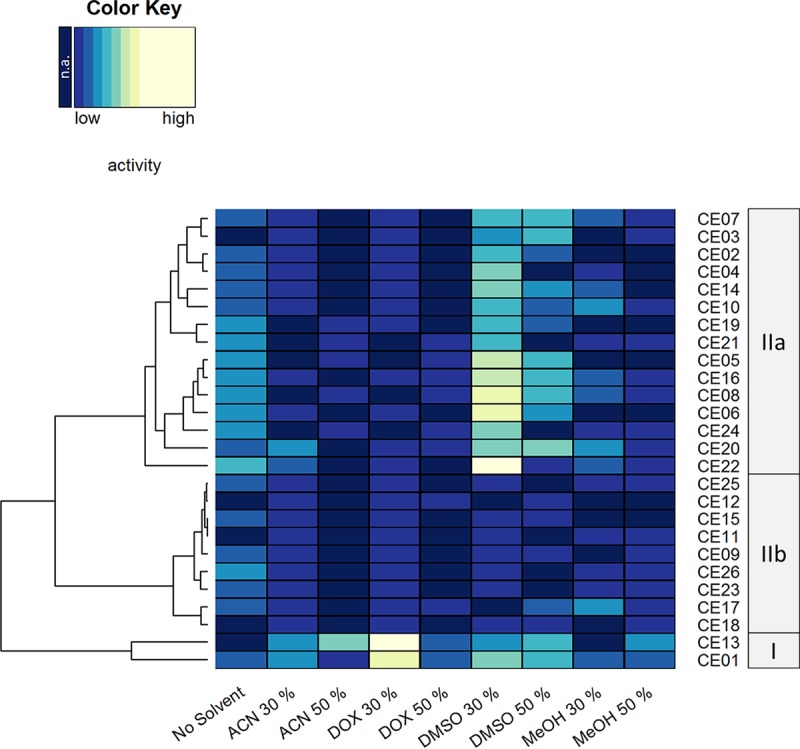
Heat map representation of CE activity in the presence of different water-miscible organic solvents. Each row represents an individual enzyme, with the enzyme identifier depicted on the right side. Columns stand for respective organic solvents indicated at the bottom. The dendrogram on the left side indicates a hierarchical clustering of CEs based on their activity in the presence of different organic solvents. CE classes of different solvent tolerance are indicated in gray boxes on the right. The activity data are visualized with dark blue (not active [n.a.]) to yellow (highly active), indicated by the color key. The conditions tested were without addition of organic solvent (no solvent), acetonitrile (ACN), 1,4-dioxane (DOX), dimethyl sulfoxide (DMSO), or methanol (MeOH) at 30% or 50% (vol/vol) concentrations. Reactions were carried out at 30°C for 18 h in 5 mM potassium phosphate buffer (pH 7.2) containing 20 μg/ml nitrazine yellow.

The activity data were plotted as a heat map, and enzymes were hierarchically clustered according to their activities in different solvent systems and visualized by a row dendrogram (Fig. 2). Three groups of enzymes could be distinguished based on their tolerance toward organic solvents, as follows: I, tolerant enzymes with prominent activity under almost all tested conditions; IIa, medium-tolerant enzymes displaying high activity when a low concentration of dimethyl sulfoxide (DMSO) was present; and IIb, sensitive enzymes showing decreased activity.

As expected, the benchmark enzyme HZ lipase (CE01) proved to be tolerant, showing activity under all tested conditions. Interestingly, CE13 from *P. aestusnigri* was found to be similarly tolerant, exhibiting even higher activity in the presence of 50% acetonitrile, which was the most disruptive reaction condition tested here. Remarkably, this enzyme did not show prominent activity without solvent added, indicating activation by organic solvents. Two enzymes, CE16 and CE20, were found to be active in all organic solvents except 50% acetonitrile, with CE20 showing higher activity than that of CE16 in the presence of 30% acetonitrile. Moreover, the majority of the enzymes were active at high concentrations of methanol and dimethyl sulfoxide but not 1,4-dioxane or acetonitrile. Activity was detected at 50% but not at 30% (vol/vol) organic solvent concentrations for CE03, CE09, and CE13 with methanol, for CE08 and CE19 with acetonitrile, for CE17 and CE12 with dimethyl sulfoxide, and for CE05, CE21, and CE24 with acetonitrile and 1,4-dioxane. This observation might reflect an activating effect of the organic solvent described for different enzymes, including lipases (31–34).

**FIG 3:**
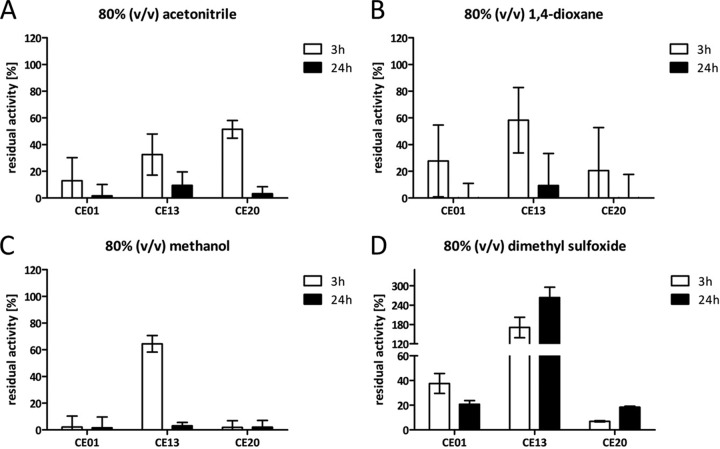
Residual CE activity after incubation in the presence of organic solvents. (A to D) Enzymes were incubated for 3 h and 24 h in 80% (vol/vol) concentration of acetonitrile (A), 1,4-dioxane (B), methanol (C), or dimethyl sulfoxide (D). Residual activity was determined with 4-nitrophenyl butyrate as the substrate and calculated relative to the initial activity of the respective enzyme set as 100%. Error bars indicate standard deviations of the results from three separate experiments. Reactions were conducted at 30°C in 100 mM potassium phosphate buffer (pH 7.2).

Based on these results, we selected enzymes CE13 and CE20 and the benchmark enzyme CE01 for further characterization. The respective cell lysates were incubated with 80% (vol/vol) organic solvents, since most enzymes are rapidly inactivated at concentrations above 70% (vol/vol) (35), and the residual activity was determined after 3 h and 24 h (Fig. 3). Under these conditions, the activity of CE01 rapidly decreased during incubation in acetonitrile, 1,4-dioxane, and methanol (Fig. 3A to C); about 38% of the residual activity was retained after 3 h and 21% after 24 h of incubation in dimethyl sulfoxide (Fig. 3D). The newly identified esterase CE13, which we proposed to be highly solvent tolerant, showed 33%, 58%, and 64% residual activity after 3 h of incubation in acetonitrile, 1,4-dioxane, and methanol, respectively (Fig. 3A to C). After 24 h of incubation, the residual activity further decreased to less than 10%. Remarkably, increased activity was observed in the presence of dimethyl sulfoxide, which resulted in about 264% activity after 24 h (Fig. 3D). CE20 appeared less resistant and showed a complete loss of activity when methanol was present and a rapid deactivation by 1,4-dioxane (Fig. 3B and C). When dimethyl sulfoxide was present for 3 h, less than 10% residual activity was measured; however, at an extended incubation time, residual activity was determined to be about 18% (Fig. 3D). Notably, about 50% residual activity was detected for CE20 after 3 h of incubation with acetonitrile, whereas no activity was left after 24 h (Fig. 3A). In contrast, no activity was observed with the nitrazine yellow assay in the presence of 50% acetonitrile, indicating that CE20 may be at least partly reactivated when the enzyme is transferred from organic to aqueous solvent.

The observation of increased enzyme activity upon incubation in organic solvents was previously connected to a temperature significantly below the enzyme’s half-inactivation temperature (*T*_50_) (34). The *T*_50_s of CE01, CE13, and CE20 were determined to be 58°C, 56°C, and 57°C, respectively (Fig. S2). These values do not differ in a range large enough to explain the observation that only CE13 was “activated” upon incubation in DMSO at an assay temperature of 30°C.

### Screening of CEs active toward substrates with poor water solubility.

**FIG 4:**
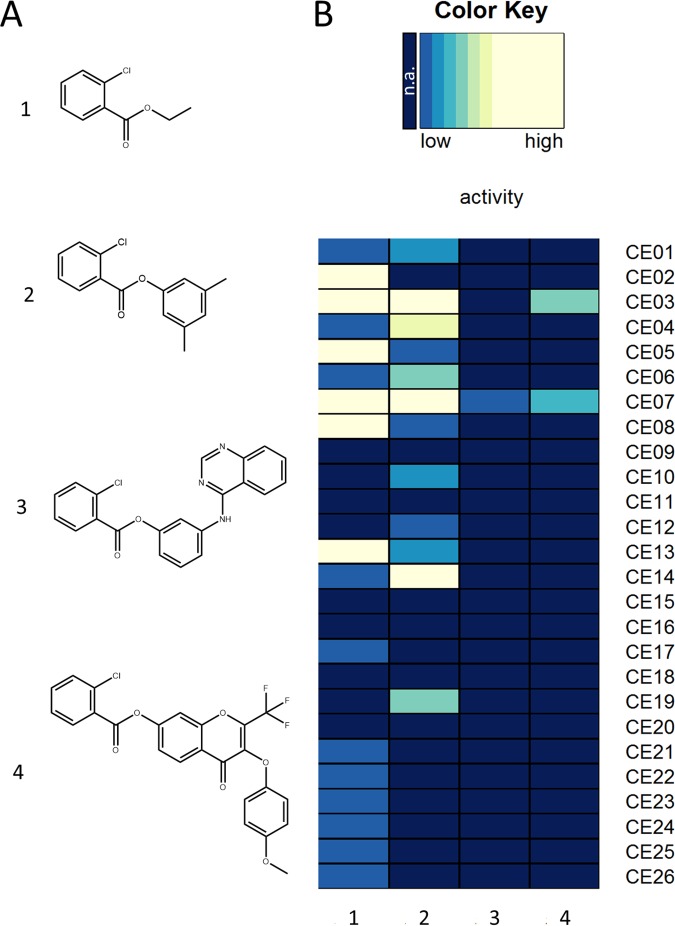
Hydrolysis of 2-chlorobenzoate esters in the presence of 30% (vol/vol) dimethyl sulfoxide determined by the nitrazine yellow assay. (A) Structural formulas of tested compounds 1 to 4. (B) Heat map plot of enzyme activities. The substrates are as follows: 1, ethyl 2-chlorobenzoate; 2, 3,5-dimethylphenyl 2-chlorobenzoate; 3, 3-(quinazolin-4-ylamino)phenyl 2-chlorobenzoate; and 4, 3-(4-methoxyphenoxy)-4-oxo-2-(trifluoromethyl)-4*H*-chromen-7-yl 2-chlorobenzoate. Each row of the heat map represents an individual enzyme with the enzyme identifier indicated on the right side. Each column represents a different substrate. The activity data are visualized from dark blue (not active [n.a.]) to yellow (highly active), as indicated by the color key. The reaction conditions were 18 h of incubation at 30°C in 5 mM potassium phosphate buffer (pH 7.2) containing 20 μg/ml nitrazine yellow, 30% (vol/vol) dimethyl sulfoxide, 5% (vol/vol) acetonitrile, and substrates 1 to 4 at a concentration of 10 mM.

The ability of CEs to accept multiple substrates is an important property for biocatalytic applications; however, many industrially relevant compounds are poorly soluble in water. We therefore decided to test the newly expressed CEs for their ability to hydrolyze sterically demanding substrates of low solubility in water using the nitrazine yellow assay and 30% (vol/vol) dimethyl sulfoxide as a cosolvent. Four different substrates of increasing complexity were used which all represent esters of 2-chlorobenzoate (2-CBA), namely, ethanol (substrate 1), xylenol (substrate 2), 3-(quinazolin-4-ylamino)phenol (substrate 3), and 3-(4-methoxyphenoxy)-4-oxo-2-(trifluoromethyl)-4*H*-chromen-7-ol (substrate 4) (Fig. 4A). The latter two compounds mimic precursor for approved drugs like the tyrosine-kinase inhibitor gefitinib, used in lung cancer treatment (36), or novel compounds that are promising for the treatment of different types of cancer (37, 38). 2-CBA is a strong carbonic acid thus enabling the detection also of enzymes with low activity, which may represent potential candidates for enzyme engineering. Remarkably, CE07 hydrolyzed all four substrates (Fig. 4B), and CE03 hydrolyzed substrates 1, 2, and 4, whereas most of the CEs tested could not hydrolyze substrates 3 or 4 (Fig. 4B and S2). Substrate 3 was not completely soluble in 30% (vol/vol) DMSO; however, enzyme activity could be determined by measuring the ratio of the absorptions at 450 and 600 nm. These results were confirmed by repeating the reactions with 5 U each of CE07 and CE03 (determined with 4-nitrophenyl butyrate as the substrate) and detection of the products by high-performance liquid chromatography (HPLC) (Fig. S3). A commercial preparation of CalB was included as a reference enzyme known to accept many structurally diverse ester substrates (17). Both CE03 and CE07 hydrolyzed all substrates, whereas CalB hydrolyzed only substrates 1 and 3 (Table 2). In this assay, in contrast to the nitrazine yellow assay, hydrolysis of compound 3 by CE03 could also be demonstrated. CE07 hydrolyzed all 4 substrates and was the best performing enzyme with substrate 3.

**TABLE 2:** Enzyme activities of CE03, CE07, and CalB toward substrates 1 to 4^*a*^

Substrate no.	Concn of 2-CBA (mM) with:							
	CE03		CE07		CalB		No enzyme	
	Mean	SD	Mean	SD	Mean	SD	Mean	SD
1	4.340	1.124	3.593	1.290	5.177	0.408	0.020	0.000
2	4.823	0.667	3.297	2.013	NA		0.033	0.019
3	1.007	0.295	4.563	0.034	0.143	0.005	0.053	0.009
4	3.740	0.120	1.983	0.581	NA		0.033	0.017

a The hydrolysis product 2-chlorobenzoate (2-CBA) was detected by HPLC and is given as mean concentration from three independent reactions along with standard deviations. A reaction mix without the addition of enzyme served as a control; CBA concentrations in the range of the control reaction indicated no activity (NA). The substrates were as follows: 1, ethyl 2-chlorobenzoate; 2, 3,5-dimethylphenyl 2-chlorobenzoate; 3, 3-(quinazolin-4-ylamino)phenyl 2-chlorobenzoate; and 4, 3-(4-methoxyphenoxy)-4-oxo-2-(trifluoromethyl)-4*H*-chromen-7-yl 2-chlorobenzoate. The reaction conditions were 30°C for 18 h with 5 U of enzyme, 5 mM substrate, 30% DMSO, and 70 mM potassium phosphate buffer (pH 7.2).

In addition to these results, we also studied the CE substrate specificity with a set of 96 chemically and structurally different ester substrates, as described recently (17, 39). In this assay system, CE07 was also identified as highly substrate promiscuous, accepting 65 different ester substrates, but CE03 exhibited only medium promiscuity, hydrolyzing 25 esters. In contrast, some enzymes proving to be highly substrate promiscuous in this assay system, e.g., CE13, which hydrolyzed 51 different esters and did show prominent activity in the presence of organic solvents, were inactive against substrates 3 and 4 (Table S2).

## DISCUSSION

Tolerance against various organic solvents and acceptance of diverse synthetic substrates are both required for applications of CEs in industrial biocatalysis. Substrate promiscuity has recently been investigated in detail (17), but tolerance against organic solvents has not been systematically investigated with a larger set of enzymes. Tolerance against organic solvents is often determined by measuring the residual activity of an enzyme after incubation but not in the presence of a solvent (40–47). The accuracy of this approach is improved by following the time-dependent decrease in enzyme activity over a longer period of time, a method that is not suitable for high-throughput screening approaches. On the other hand, a variety of pH shift assays are available that allow for a determination of enzyme activities also at high throughput (12, 39, 48). To the best of our knowledge, organic solvent tolerance was not systematically investigated using a pH shift assay. pH indicators such as 4-nitrophenol (used at pH 7.0) and phenol red (used at pH 8.0) (17, 48) support concentrations of solvents lower than 30%. Some indicator compounds, such as anilines, are known to tolerate high concentrations of organic solvents, e.g., acetonitrile (49); however, they are not suitable to detect shifts from physiological pH. In this study, we observed that the indicator dye nitrazine yellow undergoes a color shift below pH 7 (29, 50) and can thus be used for the determination of pH changes in the presence of different water-miscible organic solvents at concentrations of up to 50% (vol/vol). Notably, this approach is limited to testing of water-miscible organic solvents; nonpolar organic solvents form two-phase systems, which are difficult to read out with colorimetric microtiter plate (MTP) scale assays.

Here, we have described an assay applicable for the fast and simple determination of solvent-tolerant CEs at high throughput, which was applied to a benchmark CE and 25 CEs from *A. borkumensis* and *P. aestusnigri*, two marine oil-degrading bacteria that were shown to represent a prolific, and, in the case of *P. aestusnigri* (20), nearly unexplored, source of this class of enzymes. This observation indicates that oil-degrading bacteria may represent a prolific source for organic-solvent-tolerant enzymes. We have identified a number of organic-solvent-tolerant CEs that are also active against water-insoluble substrates mimicking industrial relevant compounds.

To describe this in greater detail, the method allowed the identification of CEs with outstanding performance in the presence of organic solvents which are commonly very harmful to the activity of these enzymes. Particularly, compared to other reported organic-solvent-tolerant CEs, CE13, identified here using the nitrazine yellow assay, can withstand organic solvents even at higher concentrations, retaining about 30% residual activity after 3 h of incubation in 80% (vol/vol) acetonitrile. For comparison, the organic-solvent-tolerant ARM lipase from Geobacillus sp. strain ARM showed a near-complete inactivation after a 30-min incubation in 30% (vol/vol) acetonitrile (47), the lipase from Staphylococcus saprophyticus M36 displayed 27% residual activity after a 30-min incubation in 25% (vol/vol) acetonitrile (42), and the cold-adapted lipase LipP from Pseudomonas sp. strain B11-1 was completely inactivated after 1 h of incubation in 30% (vol/vol) acetonitrile (51). Nevertheless, there are enzymes with reported tolerance against acetonitrile, for example, a lipase from Burkholderia ambifaria YCJ01, which retained full activity after 24 h of incubation in 25% (vol/vol) acetonitrile and about 60% residual activity after 60 days under these conditions (52). Not only this stability in the presence of acetonitrile but also the about 3-fold activation after 24 h in the presence of dimethyl sulfoxide at concentrations as high as 80% are outstanding. This solvent is very deleterious for CEs because of its highly polar character, and, to the best of our knowledge, no example of a CE that shows such an activation level has been reported to date. The general phenomenon of enzyme activation upon incubation in increasing concentrations of organic solvents was reported to be connected to a significant difference between the assay temperature and an enzyme’s thermal half-inactivation (*T*_50_) (34). This might point to a limitation of our approach, such that enzymes with a *T*_50_ significantly above 30°C were identified as organic solvent tolerant. However, none of these enzymes were found to be completely inactive, suggesting that all are stable and active at 30°C (note that substrate was added after 2 h of incubation at 30°C). Moreover, the *T*_50_s of enzymes CE01, CE13, and CE20 were determined to differ by only 2°C, suggesting that the observed differences in organic solvent tolerance were not caused by differences between the assay temperature and the *T*_50_. The screening strategy described here can thus speed up the detection of CEs with prominent organic solvent tolerance, which is regarded as an important feature for biotechnological applications of CEs.

At the same time, the method can facilitate the identification of CEs active against substrates that, because of their poor water solubility, require the addition of high concentration of deleterious solvents. The enzymes CE03 and CE07 can serve as examples, as they were found to accept sterically demanding ester substrates often present in pharmaceutically relevant compounds.

In conclusion, we have examined 26 CEs; of these, the isolation of 11 CEs has been previously reported, and 15 CEs, to the best of our knowledge, have not been reported previously. Among them, CE13 from *P. aestusnigri* shows high organic solvent tolerance, and CE03 and CE07 from *A. borkumensis* exhibit a broad substrate specificity and activity toward complex ester substrates mimicking pharmaceutical building blocks. Furthermore, a screening method with the indicator dye nitrazine yellow was established, which allows for fast and simple identification of novel organic-solvent-tolerant CEs.

## MATERIALS AND METHODS

### Construction of genomic libraries.

Small-insert genomic libraries were constructed with genomic DNA extracted from cells of Pseudomonas aestusnigri and Alcanivorax borkumensis, as described previously (53). Freeze-dried cells of the *P. aestusnigri* VGXO14 (DSM 103065) and *A. borkumensis* SK2 (DSM 11573) strains were purchased from the German Collection of Microorganisms and Cell Cultures (DSMZ, Braunschweig, Germany). *P. aestusnigri* was grown in LB broth (Luria/Miller) (Carl Roth, Karlsruhe, Germany) and *A. borkumensis* in marine broth 2216 (BD Difco, Heidelberg, Germany) supplemented with 1% (wt/vol) sodium pyruvate at 30°C for 2 days or until sufficient cell growth was observed. Cells were collected by centrifugation, and genomic DNA was extracted by chemical lysis and phenol-chloroform extraction, as described earlier (54). The genomic DNA was fragmented by sonication, and DNA fragments of 5 to 10 kb were recovered by extraction from an agarose gel with the NucleoSpin gel and PCR clean-up kit (Macherey-Nagel, Düren, Germany). The DNA fragments were end repaired with T4 DNA polymerase (Thermo Fisher Scientific, Darmstadt, Germany) and a Klenow fragment (Thermo Fisher Scientific), terminal phosphates were cleaved by FastAP (Thermo Fisher Scientific), and adenine overhangs were introduced by *Taq* DNA polymerase (Thermo Fisher Scientific). Subsequently, the DNA fragments were cloned with the TOPO XL PCR Cloning kit (Invitrogen, Solingen, Germany), as recommended by the manufacturer. Competent E. coli TOP10 cells (Thermo Fisher Scientific) were transformed with the recombinant pCR-XL-TOPO plasmid library into by electroporation. Recombinant E. coli TOP10 cells were cultivated in LB broth (Luria/Miller) (Carl Roth) and autoinduction medium (20 g/liter tryptone from casein, 5 g/liter NaCl, 5 g/liter yeast extract, 6 g/liter Na_2_HPO_4_, 3 g/liter KH_2_PO_4_, 0.6% glycerol, 0.2% lactose, 0.05% glucose) (reference 55, modified according to https://openwetware.org/wiki/Lidstrom:Autoinduction_Media) at 37 and 30°C for DNA replication and protein production, respectively.

### Activity-based screening for carboxylic ester hydrolases.

Genomic libraries from *P. aestusnigri* and *A. borkumensis* were screened using E. coli TOP10 as a host, pCR-XL-TOPO as a vector, and tributyrin-containing agar plates for the identification of esterase-producing clones, as described earlier (56). The clone libraries were plated on agar plates (LB medium, 1.5% [wt/vol] agar, 50 μg/ml kanamycin, 1.5% [vol/vol] tributyrin, and 1.5 g/liter gum arabic) and incubated for 1 day at 37°C, following incubation for up to 1 week at room temperature. Clones showing halo formation were collected and grown overnight at 37°C and 150 rpm in a 100-ml Erlenmeyer flask filled with 10 ml LB broth (Luria/Miller) (Carl Roth, Karlsruhe, Germany) supplemented with 50 μg/ml kanamycin. Esterase activity was confirmed as described previously (57), using 4-nitrophenyl butyrate (*p*NPB) as the substrate. Plasmid DNA was extracted from active clones with the innuPREP plasmid minikit 2.0 (Analytik Jena, Jena, Germany). The size of the inserted DNA fragment was determined by hydrolysis with EcoRI (Thermo Fisher Scientific, Darmstadt, Germany), followed by agarose gel electrophoresis. The terminal ends of the insert DNA were Sanger sequenced (by Eurofins Genomics, Ebersberg, Germany) using the oligonucleotides included in the TOPO XL PCR Cloning kit (Invitrogen, Solingen, Germany). The resulting sequences were mapped to genomes of *P. aestusnigri* (RefSeq accession no. NZ_NBYK00000000.1) or *A. borkumensis* (RefSeq accession no. NC_008260.1) to identify the complete insert sequence of the corresponding DNA fragment. To identify CE-encoding genes, insert DNA sequences were analyzed by searching GenBank and using the ORFfinder (58) and BASys annotation (59) tools. The gene encoding the HZ lipase from Aneurinibacillus thermoaerophilus strain HZ (designated CE01) was amplified from a metagenomic library clone (A. Bollinger, S. Thies, R. Koch, and K.-E. Jaeger, unpublished data). For high-level expression of selected CEs, genes were PCR amplified with Phusion high-fidelity DNA polymerase (Thermo Fisher Scientific), following the manufacturer’s recommendations using specific oligonucleotides (Table 3), and subsequently cloned into pET-22b(+) vector (Novagen, Darmstadt, Germany) by sequence- and ligase-independent cloning (60) or directional cloning using restriction and ligation (61) with the endonuclease NdeI, XbaI in combination with XhoI, or HindIII (Thermo Fisher Scientific).

**TABLE 3:** Oligonucleotides used for PCR amplification and cloning of CEs identified by naive screening^*a*^

Enzyme ID	Oligonucleotide sequence (5′→3′)	
	Forward	Reverse
CE01	CTTTAAGAAGGAGATATACATATGCAAAAGGAAAGAAAAAATC	CAGTGGTGGTGGTGGTGGTGCTCTCTCACAGATAATGAACC
CE02	GCTCATATGAATCCTGCCGTTATTGAG	TACCTCGAGCAACCGCCGCTTGGTCTCAAC
CE03	CTTTAAGAAGGAGATATACATATGGCTTCTATTCCCGCAC	GTGGTGGTGGTGGTGGTGCTCTGACGATATCTCCGGGATTG
CE07	GTCCATATGAGCCTTCAAGCCCG	TACCTCGAGTGCTTCTTTAATGAATGCGACAATC
CE13	GCGCATATGCCTCAATCTTTTAAAC	CTTCTCGAGGGGCAATACCAGCGGCG
CE14	CTTTAAGAAGGAGATATACATATGAGCGGACTCAACCGG	CAGTGGTGGTGGTGGTGGTGCTCGCTGAGCGTCGGCACCAG
CE15	GCGCATATGTCCAGGTACGTTGATG	CGCCTCGAGGCTTACCGAGTCGGCCTG

a Restriction endonuclease sites used for directional cloning are underlined; oligonucleotides without a marked restriction site were used for sequence- and ligase-independent cloning (SLIC).

### Sequence-based screening and cloning of esterases.

In addition to CE genes identified by activity-based screening, CE genes were identified by a text search (search term “lipase,” “carboxylesterase,” or “esterase”) of the GenBank file containing the reference sequences for *P. aestusnigri* (RefSeq accession no. NZ_NBYK00000000.1) and *A. borkumensis* (RefSeq accession no. NC_008260.1). The respective genes were cloned into pET-22b(+) (Novagen, Darmstadt, Germany), as described above, using specific oligonucleotides (Table 4).

**TABLE 4:** Oligonucleotides used for PCR amplification and cloning of CEs identified by a genome sequence search^*a*^

Enzyme ID	Oligonucleotide sequence (5′→3′)	
	Forward	Reverse
CE09	GAGCATATGAGCCTGTTTGTTGATCGCATCAG	GCGAAGCTTTCATGCGTGAGCGTCCTCTTC
CE10	CGCATATGGATCTGATCATTTTTCTGC	CGGAAGCTTGTTGCAGATCAATATTTAC
CE11	ATACATATGCCGGTCCCCGAAAC	GACAAGCTTTCAGGCGTGTATTTCAATC
CE12	GCGCATATGGAACCACTTGAACTTGAGGAC	GCGAAGCTTCTATTCACTCAGGTAGCTGAGCACAAC
CE16	AGGTCTAGATGGAGGCTACACCTCATG	GTGCTCGAGGTACGGGCAGTTGCCGCGATAATC
CE17	GCGCATATGCACACTCTGTTCAAACG	GCGAAGCTTTCAGTCCAAGGCCTGC
CE18	GCGCATATGAATAACCTTACGTTACTGCCC	GACAAGCTTCGCTTGCGCTTCCAGCC
CE19	GCGCATATGGTGGTCAATCTCTTTCAGC	GACAAGCTTCGCTTTTTCCCAACCGCGTG
CE20	GCGCATATGTCACCGCAC	GACAAGCTTCGCAAGTCCGAGGCGTTC

a Restriction endonuclease sites used for directional cloning are underlined.

### Expression of carboxylic ester hydrolases.

CE-producing strains E. coli BL21(DE3) (62) carrying pET-22b(+) and E. coli TOP10 carrying pCR-XL-TOPO were grown in triplicate for 24 h at 37°C and 800 rpm in deep-well plates with 1 ml LB broth (Luria/Miller) (Carl Roth, Karlsruhe, Germany) supplemented with the appropriate antibiotic and 0.5% glucose. Twenty microliters of these cultures was used to inoculate expression cultures in 980 μl autoinduction medium (20 g/liter tryptone from casein, 5 g/liter NaCl, 5 g/liter yeast extract, 6 g/liter Na_2_HPO_4_, 3 g/liter KH_2_PO_4_, 0.6% glycerol, 0.2% lactose, 0.05% glucose) (reference 55, modified according to https://openwetware.org/wiki/Lidstrom:Autoinduction_Media) with the respective antibiotic, and were incubated for 20 h at 30°C under agitation at 800 rpm. The cultures were harvested by centrifugation and the supernatants discarded, and the cells were suspended in 100 μl cell lysis solution containing polymyxin B (10 mM potassium phosphate buffer [pH 7.2], 0.1 mg/ml polymyxin B) and incubated for 1 h at 37°C.

### Amino acid sequence analysis of carboxylic ester hydrolases.

The amino acid sequences of CEs used in this study were aligned with a set of enzymes representing known examples of each family of bacterial lipolytic enzymes (25). The alignment was performed using Clustal Omega (63), the phylogenetic tree was constructed with IQ-TREE (64), under default conditions, and graphical representation was done using iTOL (65). The global sequence identity matrix was obtained using Clustal Omega multiple-sequence alignment with the amino acid sequences of the CEs used in this study.

### Nitrazine yellow assay to determine organic solvent tolerance.

The organic solvent tolerance of CEs was determined by mixing 100 μl of the CE-containing cell extracts with 100 μl of the respective solvent in a microtiter plate (MTP) to reach final solvent concentrations of 0%, 30%, and 50% (vol/vol) and incubation for 2 h at 30°C. During incubation, the MTP lid was sealed with organic-solvent-stable tape to prevent evaporation. After preincubation with organic solvents, 10 μl of the sample was combined with 180 μl nitrazine yellow-containing assay buffer (5 mM potassium phosphate buffer [pH 7.2], 20 μg/ml nitrazine yellow, and 0%, 30%, or 50% [vol/vol] of the respective organic solvent) and 10 μl of substrate solution (200 mM tributyrin in acetonitrile) or 10 μl acetonitrile for the control. In the case of a color shift after addition of the organic solvent, the pH was titrated to neutral (blue color) with potassium hydroxide solution. The reaction mixture was incubated for 18 h at 30°C and afterwards measured for pH change. Activity was measured using a Tecan infinite M1000 Pro photometer at the absorption maxima of the indicator dye (λ) of 450 and 600 nm. The quotient of the absorption values determined at both wavelengths was used to measure the pH shift. Each value was corrected by subtraction of the control which did not contain substrate before calculation of mean values and standard deviations. To reduce false positives, values in the range of the standard deviation of the empty vector control were considered not active (NA).

### Heat map plot.

The language R and the package gplots function were used to write a script allowing us to plot the activity data obtained from the nitrazine yellow assay in the form of a heat map. The code for generating the heat map is given in the Supporting Method in the supplemental material.

### Determination of organic solvent tolerance.

The CE-producing E. coli BL21(DE3) cells carrying the pET-22b(+) vector and E. coli TOP10 cells carrying the pCR-XL-TOPO vector were grown for 24 h at 37°C and 150 rpm in 100-ml Erlenmeyer flasks with 10 ml LB medium supplemented with the appropriate antibiotics and 0.5% glucose. The expression cultures were inoculated in 250-ml Erlenmeyer flasks with 25 ml autoinduction medium (20 g/liter tryptone from casein, 5 g/liter NaCl, 5 g/liter yeast extract, 6 g/liter Na_2_HPO_4_, 3 g/liter KH_2_PO_4_, 0.6% glycerol, 0.2% lactose, 0.05% glucose) (reference 55, modified according to https://openwetware.org/wiki/Lidstrom:Autoinduction_Media) with antibiotic to an optical density at λ of 580 nm of 0.05 and incubated for 20 h at 30°C under agitation at 160 rpm. The main cultures were collected by centrifugation, the supernatant was discarded, and the cells were suspended in 1/10 of the original volume with 100 mM potassium phosphate buffer (pH 7.2). Cells were lysed by sonication, and the cell suspension was tested for esterase activity using 4-nitrophenyl butyrate as the substrate (57) and diluted accordingly. The cell suspension was mixed with 80% (vol/vol) of the organic solvents 1,4-dioxane, acetonitrile, methanol, or dimethyl sulfoxide and incubated at 30°C. After 0, 3, and 24 h of incubation, 20 μl of the solution was mixed with 180 μl assay solution (100 mM potassium phosphate buffer [pH 7.2], 1 mM 4-nitrophenyl butyrate, 5% [vol/vol] acetonitrile), and esterase activity was determined at a λ of 410 nm at 30°C for 10 min using a Tecan infinite M1000 Pro photometer.

### Determination of activity toward water-insoluble substrates.

CEs were produced as mentioned above and tested using the nitrazine yellow assay, as described above, with the following modifications: the enzymes were tested in the presence of 30% (vol/vol) DMSO without preincubation, and the substrates were no. 1, ethyl 2-chlorobenzoate; no. 2, 3,5-dimethylphenyl 2-chlorobenzoate; no. 3, 3-(quinazolin-4-ylamino)phenyl 2-chlorobenzoate; and no. 4, 3-(4-methoxyphenoxy)-4-oxo-2-(trifluoromethyl)-4*H*-chromen-7-yl 2-chlorobenzoate (kindly provided by Bayer AG, Leverkusen, Germany). The heat map was calculated and plotted as described above.

### Measurement of half-inactivation temperature.

The thermostabilities of CE01, CE13, and CE20 were investigated by measuring the enzyme half-inactivation temperatures (*T*_50_s). The enzymes were produced with E. coli LOBSTR cells (66) carrying the respective recombinant pET-22b(+) vector. The expression cultures were inoculated from precultures in 5,000-ml Erlenmeyer flasks with 500 ml autoinduction medium, as described above, and incubated for 24 h at 30°C (CE01), 25°C (CE13), or 37°C (CE20) at 160 rpm. The cultures were collected by centrifugation (30 min at 6,000 × *g* and 4°C), the supernatant was discarded, and the cells were stored at –20°C.

For protein purification, cells were suspended in purification buffer (20 mM Na_2_HPO_4_ [pH 7.4], 500 mM NaCl, 10 mM imidazole) at 10% (wt/vol) and lysed with a high-pressure homogenizer (EmulsiFlex-C5; Avestin Europe, GmbH) with three passages at 8,000 lb/in^2^. The soluble protein fraction was obtained by centrifugation (30 min, 4°C, 36,000 × *g*) and passed through 2.5 ml equilibrated nickel-nitrilotriacetic acid (Ni-NTA) matrix (Superflow; Qiagen GmbH) by gravity flow. After washing with at least 10 column volumes (CV) of purification buffer, bound proteins were eluted with 8 ml of elution buffer (20 mM Na_2_HPO_4_ [pH 7.4], 500 mM NaCl, 500 mM imidazole). The elution fraction was concentrated by centrifugal ultrafiltration (Vivaspin 20, 10,000 molecular weight cutoff [MWCO]; Sartorius AG) prior to the buffer exchange to 100 mM potassium phosphate buffer (pH 7.2) and 100 mM NaCl by using PD-10 desalting columns (GE Healthcare), according to the manufacturer’s recommendations. The purified protein fractions were stored at –20°C.

The enzyme half-inactivation temperatures were determined using enzyme solutions diluted with 100 mM potassium phosphate buffer (pH 7.2) to an activity of about 1 U/ml measured with 4-nitrophenyl butyrate as the substrate, incubated in a PCR plate, sealed with adhesive aluminum foil, and incubated at various temperatures (40 to 80°C) for 1 h using a Biometra TAdvanced gradient thermocycler (Analytik Jena, Jena, Germany). Subsequently, residual enzyme activity was measured with 4-nitrophenyl butyrate as the substrate (57). The data obtained from three reactions were plotted (mean and standard deviation) using Prism (GraphPad Software, Inc., USA). A nonlinear fit (Boltzmann sigmoidal) was used to calculate the half-inactivation temperatures.

### Detection of 2-chlorobenzoate by HPLC.

After determination of esterase activity with *p*NPB, as described previously (57), 5 U of the respective enzyme was mixed with substrate solution to give a final concentration of 5 mM the compounds 1 to 4, 70 mM potassium phosphate buffer (pH 7.2), and 30% (vol/vol) dimethyl sulfoxide as the cosolvent in polytetrafluoroethylene (PTFE)-capped glass vials. The reaction mixtures were incubated for 18 h at 30°C. Subsequently, the mixes were filtered through 0.22-μm-pore-size PTFE filters and analyzed for 2-chlorobenzoate (2-CBA) by HPLC, performed as described previously (67), using an Accucore C_18_ LC column (100 mm by 2.1 mm, 2.6-μm particle size, 80-Å pore size; Thermo Scientific) on an LC10-Ai LC system (Shimadzu, Duisburg, Germany), with a gradient of water/acetonitrile (solvent A is water with 0.1% formic acid, and solvent B is acetonitrile with 0.1% formic acid; the gradient was started at 5% B with a hold at 5% B for 1.5 min; a gradient from 5% B to 98% B for 5.5 min; a hold at 98% B for 2 min; a gradient from 98% B to 5% B in 0.5 min; and a hold at 5% B for 2 min to reequilibrate) at a flow rate of 1 ml/min. The retention time of 2-CBA was determined as 4.78 min using a pure standard. The integral of the respective signal was used to quantify the amount of 2-CBA released from the substrates based on the calibration line from a log serial dilution of 2-CBA.

### Determination of substrate specificity.

Aside from esters 1 to 4, an additional set of 96 esters with different degree of solubility were also tested to evaluate the degree of substrate promiscuity. The specific activity (units mg^−1^) determinations were assayed at 550 nm using a pH indicator (phenol red; ε_550_, 8,450 M^−1 ^cm^−1^) assay at 550 nm in 384-well plates, as previously described (17, 39). Briefly, cells were grown overnight at 37°C on solid agar medium containing inducer and antibiotics. Cells were washed from the plates, collected by centrifugation, and lysed by sonication after mixing in a vortex for 1 min in 5 mM *N*-(2-hydroxyethyl) piperazine *N*′-(3-propanesulfonic acid) buffer (EPPS buffer) adjusted to pH 8.0 with NaOH. The lysed cells were combined with 96 different esters as the substrates and phenol red as a pH indicator in 384-well plates, giving a final concentration of 1.14 mg/ml the respective ester, 0.45 mM phenol red, 4.5% acetonitrile, and about 1 mg/ml lysed cells in 44 μl EPPS buffer (pH 8.0). Reaction mixtures were incubated at 30°C, and hydrolysis was followed at 550 nm for 24 h to calculate specific enzyme activities. Calculations were performed in triplicate and corrected for nonenzymatic transformation.

## Supplementary Material

Supplemental file 1

Supplemental file 2
