# Supplementary material for: Organic-Solvent-Tolerant Carboxylic Ester Hydrolases for Organic Synthesis
Source: Appl Environ Microbiol. 2020 Apr 17;86(9):e00106-20. doi: 10.1128/AEM.00106-20 (PMC7170478; doi:10.1128/AEM.00106-20)
Supplement: Supplemental file 1 [file AEM.00106-20-s0001.pdf]

Supplementary Material

Identification of organic solvent tolerant carboxylic ester hydrolases for organic synthesis

Alexander Bollinger[1a], Rebecka Molitor[1a], Stephan Thies[1], Rainhard Koch[2], Cristina Coscolín[3], Manuel Ferrer[3] and Karl-Erich Jaeger[1,4*]

[1] Institute of Molecular Enzyme Technology, Heinrich Heine University Duesseldorf, Juelich, Germany

[2] Bayer AG, Leverkusen, Germany

[3] Institute of Catalysis, Consejo Superior de Investigaciones Científicas, Madrid, Spain

[4] Institute for Bio- and Geosciences IBG-1: Biotechnology, Forschungszentrum Jülich GmbH, Juelich, Germany

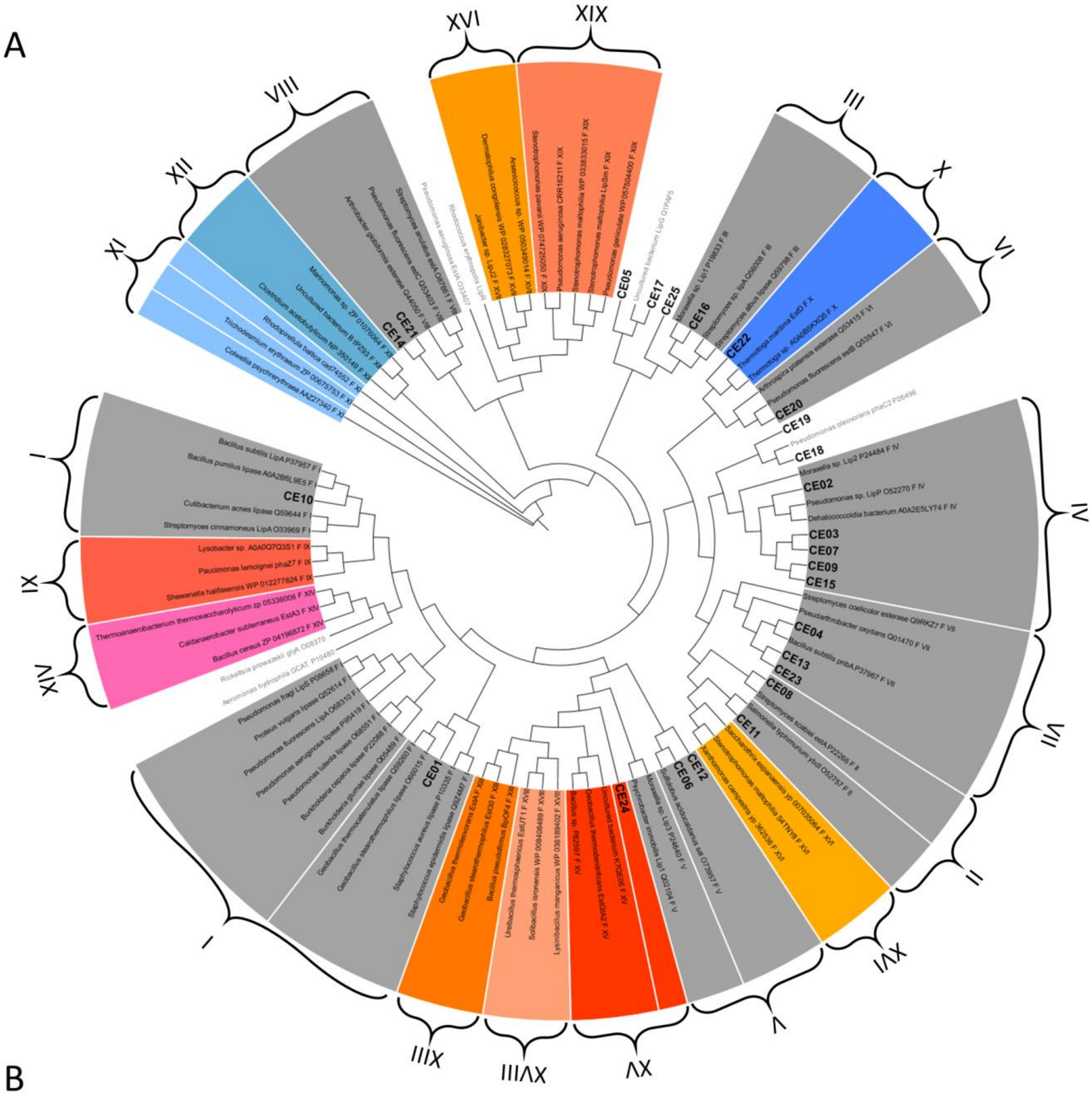

B

	CE01	CE02	CE03	CE04	CE05	CE06	CE07	CE08	CE09	CE10	CE11	CE12	CE13	CE14	CE15	CE16	CE17	CE18	CE19	CE20	CE21	CE22	CE23	CE24	CE25
**CE01**	100	12	12	17	13	20	13	15	17	17	13	12	18	14	16	17	11	14	15	11	10	9	19	10	18
**CE02**	12	100	26	14	12	12	24	10	27	11	14	11	12	6	29	11	16	14	16	20	6	15	12	12	16
**CE03**	12	26	100	12	15	8	46	13	31	13	13	11	10	5	35	9	10	16	16	21	9	13	11	14	13
**CE04**	17	14	12	100	13	19	14	16	12	12	21	15	31	17	18	13	11	13	17	17	15	13	31	11	15
**CE05**	13	12	15	13	100	10	15	14	13	12	10	12	12	13	15	11	26	13	16	11	12	17	16	13	14
**CE06**	20	12	8	19	10	100	13	15	12	11	16	14	16	21	11	15	9	6	6	13	15	11	18	11	13
**CE07**	13	24	46	14	15	13	100	11	29	14	16	12	9	4	29	11	10	17	16	21	6	15	13	15	14
**CE08**	15	10	13	16	14	15	11	100	8	10	23	17	18	14	9	9	11	7	10	7	14	13	18	4	12
**CE09**	17	27	31	12	13	12	29	8	100	18	18	7	11	8	43	14	10	15	15	23	8	14	13	14	11
**CE10**	17	11	13	12	12	11	14	10	18	100	16	14	15	7	16	17	9	12	18	16	6	21	14	18	17
**CE11**	13	14	13	21	10	16	16	23	18	16	100	29	14	12	22	22	4	10	13	17	11	9	16	13	11
**CE12**	12	11	11	15	12	14	12	17	7	14	29	100	12	7	9	19	14	8	16	14	12	9	15	5	18
**CE13**	18	12	10	31	12	16	9	18	11	15	14	12	100	14	14	19	13	11	13	11	15	14	41	11	16
**CE14**	14	6	5	17	13	21	4	14	8	7	12	7	14	100	8	13	10	12	9	3	38	9	14	6	16
**CE15**	16	29	35	18	15	11	29	9	43	16	22	9	14	8	100	13	11	16	18	24	11	15	14	13	13
**CE16**	17	11	9	13	11	15	11	9	14	17	22	19	19	13	13	100	7	12	18	10	12	13	16	13	30
**CE17**	11	16	10	11	26	9	10	11	10	9	4	14	13	10	11	7	100	16	20	13	7	10	14	5	18
**CE18**	14	14	16	13	13	6	17	7	15	12	10	8	11	12	16	12	16	100	13	17	11	14	9	14	11
**CE19**	15	16	16	17	16	6	16	10	15	18	13	16	13	9	18	18	20	13	100	17	11	20	14	17	14
**CE20**	11	20	21	17	11	13	21	7	23	16	17	14	11	3	24	10	13	17	17	100	4	15	15	15	16
**CE21**	10	6	9	15	12	15	6	14	8	6	11	12	15	38	11	12	7	11	11	4	100	10	13	7	12
**CE22**	9	15	13	13	17	11	15	13	14	21	9	9	14	9	15	13	10	14	20	15	10	100	12	15	12
**CE23**	19	12	11	31	16	18	13	18	13	14	16	15	41	14	14	16	14	9	14	15	13	12	100	12	16
**CE24**	10	12	14	11	13	11	15	4	14	18	13	5	11	6	13	13	5	14	17	15	7	15	12	100	8
**CE25**	18	16	13	15	14	13	14	12	11	17	11	18	16	16	13	30	18	11	14	16	12	12	16	8	100

Figure S1 Classification of CEs used in this study into the currently 19 families of bacterial lipolytic enzymes as shown by the unrooted phylogenetic tree (A). Enzymes used in this study (CE01 to CE25) are printed in bold, families I to VIII are shown in grey, whereas families IX to XIX are shown in different colors. The global sequence identity matrix of amino acid sequences of the enzymes used in this study is given as a table (B). Maximum identity (100 %) is shown by dark grey, high identity (>30 %) is shown by light grey background color.

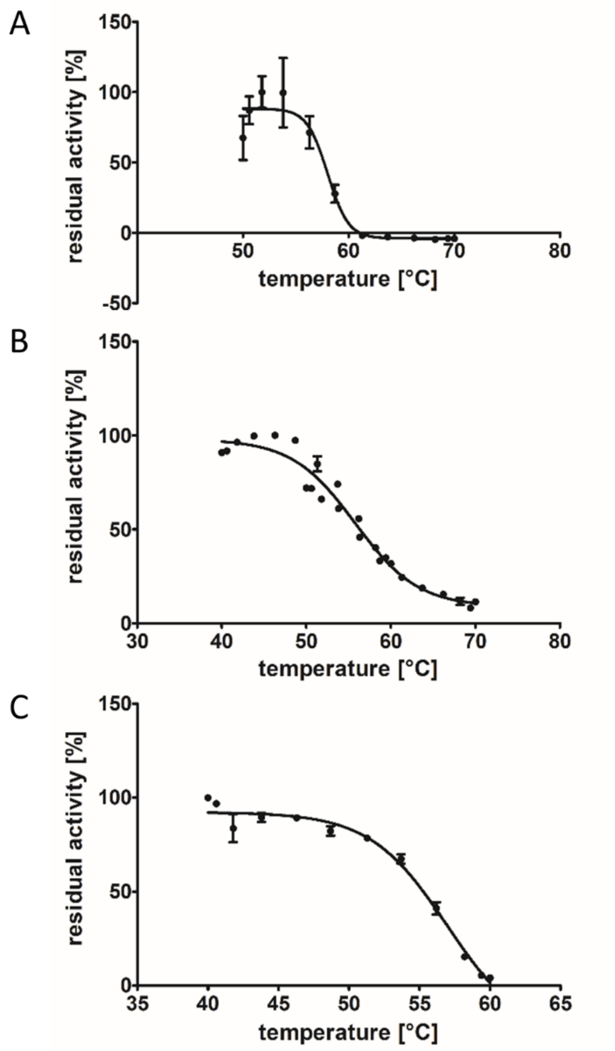

Figure S2 Thermal inactivation curves for CE01 (A), CE13 (B), and CE20 (C). Purified enzymes (about 1 U/ml in 100 mM potassium phosphate buffer pH 7.2) were incubated at various temperatures for 1 h using a PCR gradient cycler. Subsequently, enzyme activity was measured using 4-nitrophenyl butyrate as a substrate. The activity data was plotted relative to the highest activity and fitted nonlinear (Boltzmann sigmoidal). The enzymes half-inactivation temperature (T_{50}) is found at the temperature where the enzyme shows 50 % residual activity. The mean value and standard deviation of three separate reactions is shown.

	1	2	3	4	5	6	7	8	9	10	11	12
**A**	CE01	CE02	CE03	CE09	CE07	CE10	CE11	CE12	CE13	CE14	CE15	CE16
**B**	CE17	CE18	CE19	CE20	CE01	CE02	CE03	CE09	CE07	CE10	CE11	CE12
**C**	CE13	CE14	CE15	CE16	CE17	CE18	CE19	CE20	CE01	CE02	CE03	CE09
**D**	CE07	CE10	CE11	CE12	CE13	CE14	CE15	CE16	CE17	CE18	CE19	CE20
**E**	control	control	control									
**F**	CE21	CE14	CE22	CE23	CE24	CE26	CE25	CE04	CE06	CE05	CE08	CE21
**G**	CE14	CE22	CE23	CE24	CE26	CE25	CE04	CE06	CE05	CE08	CE21	CE14
**H**	CE22	CE23	CE24	CE26	CE25	CE04	CE06	CE05	CE08			

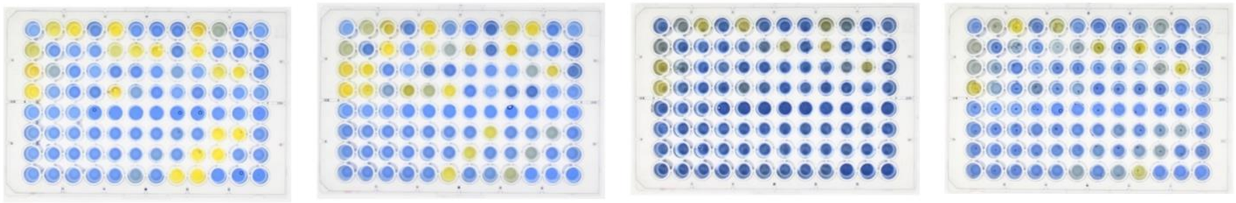

#1 #2 #3 #4

Figure S3 Layout of the assay plates and photographs after 18 h incubation at 30°C in 5 mM potassium phosphate buffer pH 7.2 containing 20 µg/ml nitrazine yellow, 30 % (v/v) dimethyl sulfoxide, 5 % (v/v) acetonitrile and 10 mM of the substrates #1 - #4. Substrates are #1: ethyl 2-chlorobenzoate. #2: 3,5-dimethylphenyl 2-chlorobenzoate. #3: 3-(quinazolin-4-ylamino)phenyl 2-chlorobenzoate. #4: 3-(4-methoxyphenoxy)-4-oxo-2-(trifluoromethyl)-4*H*-chromen-7-yl 2-chlorobenzoate.

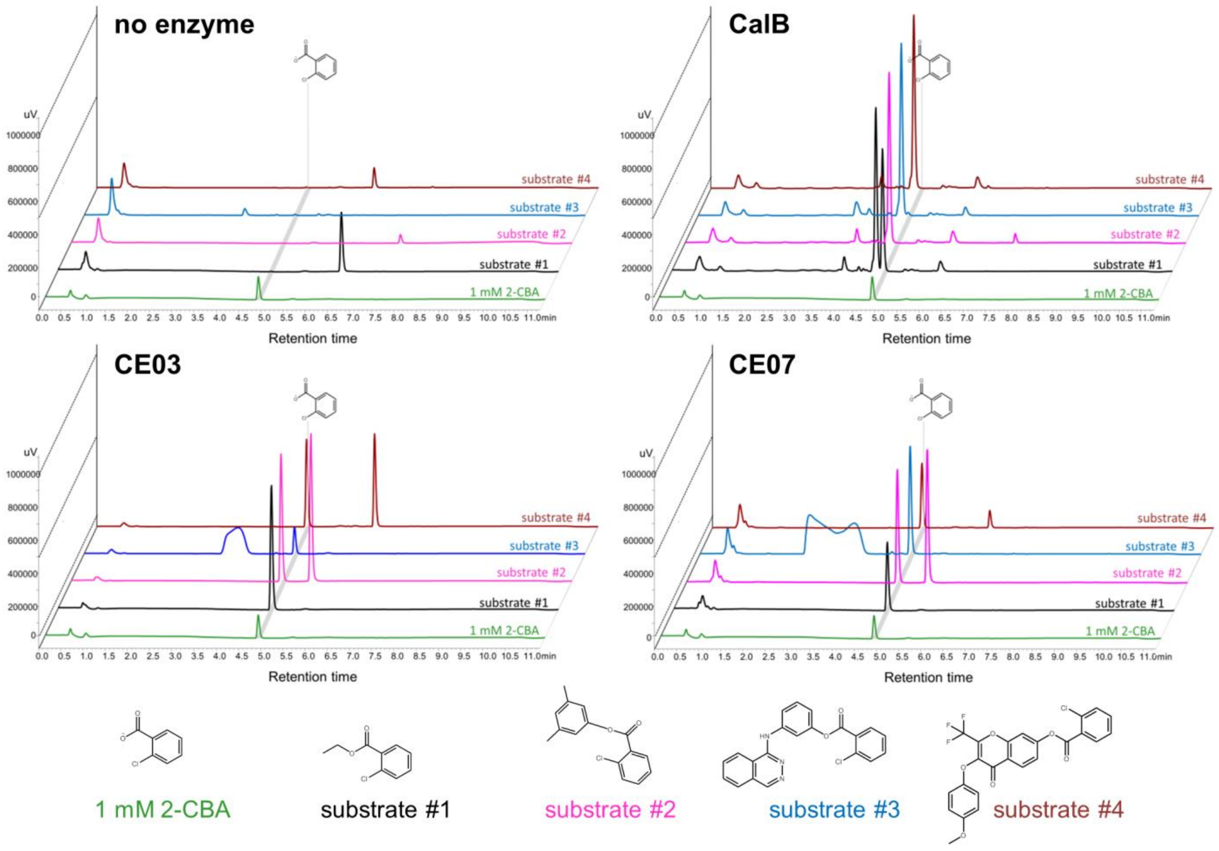

	Ret. Time	Area	Height	calc. conc. (mM)	Accuracy[%]
2-CBA Standard 0 mM	-----	-----	-----	-----	-----
2-CBA Standard 0.01 mM	4.79	4496	1335	0.01	101.8
2-CBA Standard 0.1 mM	4.79	50195	14052	0.10	99.8
2-CBA Standard 1 mM	4.79	509201	138318	1.00	100
no enzyme control #1	4.79 ±0.00	9336 ±955	2674 ±276	0.02 ±0.00	
no enzyme control #2	4.80 ±0.00	8623 ±1348	1636 ±28	0.02 ±0.00	
no enzyme control #3	4.79 ±0.00	29606 ±232	6976 ±16	0.06 ±0.00	
no enzyme control #4	4.79 ±0.00	22000 ±195	5216 ±49	0.04 ±0.00	
CalB #1	4.80 ±0.00	2638222 ±207048	714066 ±54703	5.18 ±0.41	
CalB #2	-----	-----	-----	-----	
CalB #3	4.79 ±0.01	71727 ±410876	18989 ±101753	0.14 ±0.81	
CalB #4	-----	-----	-----	-----	
CE03 #1	4.79 ±0.00	2211501 ±571791	604228 ±154686	4.34 ±1.12	
CE03 #2	4.79 ±0.00	2458861 ±340285	680460 ±91858	4.82 ±0.67	
CE03 #3	4.79 ±0.00	511791 ±150794	135939 ±38089	1.01 ±0.29	
CE03 #4	4.78 ±0.00	1907004 ±62544	534513 ±15852	3.74 ±0.12	
CE07 #1	4.79 ±0.00	1832990 ±657330	495309 ±174573	3.59 ±1.29	
CE07 #2	4.79 ±0.01	1678834 ±1026990	460839 ±280794	3.30 ±2.01	
CE07 #3	4.79 ±0.00	2326591 ±18145	619761 ±16245	4.56 ±0.03	
CE07 #4	4.78 ±0.00	1009735 ±269201	282854 ±82030	1.98 ±058	
Average (N=44)	4.789 ±0.0055				
%RSD (N=44)	0.115796				
Maximum	4.801				
Minimum	4.776				

Figure S4 Quantification of 2-Chlorobenzoic acid (2-CBA) released from different ester compounds upon enzymatic hydrolysis by CE03, CE07, or CalB. Substrates were #1: ethyl 2-chlorobenzoate. #2: 3,5-dimethylphenyl 2-chlorobenzoate. #3: 3-(quinazolin-4-ylamino)phenyl 2-chlorobenzoate. #4: 3-(4-methoxyphenoxy)-4-oxo-2-(trifluoromethyl)-4*H*-chromen-7-yl 2-chlorobenzoate. Reaction conditions were 30°C, 18 h, 5 U of enzyme, 5 mM substrate, 30 % DMSO, 70 mM potassium phosphate buffer pH 7.2.

Table S1 Specification of the genome fragment carried by the recombinant pCR-XL-TOPO plasmids of the genomic library clones used in this study with given start and end position of the fragment on respective contig (genbank id), enzyme identifier (id.), and protein accession number (acc.).

id.	acc.	vector	genbank id	start pos.	end pos.
CE04	WP_011588534.1	pCR-XL-TOPO	NC_008260.1	1424579	1428511
CE05	WP_011589376.1	pCR-XL-TOPO	NC_008260.1	2402770	2406029
CE06	WP_011589723.1	pCR-XL-TOPO	NC_008260.1	2771474	2777305
CE08	WP_011589970.1	pCR-XL-TOPO	NC_008260.1	3048294	3054618
CE21	WP_088273788.1	pCR-XL-TOPO	NBYK01000001.1	742906	734589
CE22	SEG59772.1	pCR-XL-TOPO	NBYK01000011.1	96693	107328
CE23	WP_088274564.1	pCR-XL-TOPO	NBYK01000003.1	149164	155945
CE24	WP_088275865.1	pCR-XL-TOPO	NBYK01000006.1	120599	125419
CE25	WP_088273867.1	pCR-XL-TOPO	NBYK01000001.1	836589	847776
CE26	n.d.	pCR-XL-TOPO	NBYK01000001.1	282473	286927

Table S2 Substrate promiscuity data of CEs used in this study assessed with 96 different ester substrates. Activity data are given in U/g of wet cell weight.

The dimensions of Table S2 did not allow it to be included here; Table S2 can be found as separate file in excel format.

Supporting method

The source code used to plot the heatmap in the language R is given below:

```
heatmap.2(
      matrix,
      Rowv=TRUE,
      Colv=NULL,
      col=rev(c("#FFFFDD","#FFFFDD","#FFFFDD","#FFFFDD","#FFFFDD","#FFFFDD","#
      EDF8B1","#C7E9B4","#7FCDBB","#41B6C4", "#1D91C0","#225EA8","#253494")),
      scale="none",
      margins=c(6,6),
      symm=FALSE,
      vline=NULL,
      hline=NULL,
      trace="none",
      density.info="none",
      srtCol=35,
      cexCol=0.90,
      symkey=FALSE,
      symbreaks=FALSE,
      colsep=c(1:50),
      rowsep=c(1:50),
      sepcolor="black",
      sepwidth=c(0.01,0.01),
      na.color="#081D58",
      key.xlab="activity",
      key.xtickfun=function()
      {cex<-par("cex")*par("cex.axis")
              side<-1
              line<-0
              col<-par("col.axis")
              font<-par("font.axis")
              mtext("low", side=side, at=0, adj=0,
              line=line, cex=cex, col=col, font=font)
              mtext("high", side=side, at=1, adj=1,
              line=line, cex=cex, col=col, font=font)
              return(list(labels=FALSE, tick=FALSE))})
```
